# Conveying Intention by Motions With Awareness of Information Asymmetry

**DOI:** 10.3389/frobt.2022.783863

**Published:** 2022-02-16

**Authors:** Yosuke Fukuchi, Masahiko Osawa, Hiroshi Yamakawa, Tatsuji Takahashi, Michita Imai

**Affiliations:** ^1^ Faculty of Science and Technology, Keio University, Yokohama, Japan; ^2^ Nihon University, Tokyo, Japan; ^3^ The Whole Brain Architecture Initiative, Tokyo, Japan; ^4^ School of Engineering, University of Tokyo, Tokyo, Japan; ^5^ RIKEN Center for Advanced Intelligence Project, Tokyo, Japan; ^6^ School of Science and Engineering, Tokyo Denki University, Tokyo, Japan

**Keywords:** Bayesian theory of mind, public self-awareness, PublicSelf model, human-agent collaboration, legible motion, reinforcement learning, explainable AI

## Abstract

Humans sometimes attempt to infer an artificial agent’s mental state based on mere observations of its behavior. From the agent’s perspective, it is important to choose actions with awareness of how its behavior will be considered by humans. Previous studies have proposed computational methods to generate such publicly self-aware motion to allow an agent to convey a certain intention by motions that can lead a human observer to infer what the agent is aiming to do. However, little consideration has been given to the effect of information asymmetry between the agent and a human, or to the gaps in their beliefs due to different observations from their respective perspectives. This paper claims that information asymmetry is a key factor for conveying intentions with motions. To validate the claim, we developed a novel method to generate intention-conveying motions while considering information asymmetry. Our method utilizes a Bayesian public self-awareness model that effectively simulates the inference of an agent’s mental states as attributed to the agent by an observer in a partially observable domain. We conducted two experiments to investigate the effects of information asymmetry when conveying intentions with motions by comparing the motions from our method with those generated without considering information asymmetry in a manner similar to previous work. The results demonstrate that by taking information asymmetry into account, an agent can effectively convey its intention to human observers.

## 1 Introduction

Theory of mind is the ability to infer other people’s mental states, such as their beliefs, desires, and intentions, from their actions. By attributing mental states to others, people attempt to interpret their past behavior and predict their future actions (Premack and Woodruff, 1978). The ability to infer others’ minds in this way serves as a basis for social interaction (Marchesi et al., 2019). In cooperation, for example, a worker requires mutual understanding of what another worker is intending to do to decide how to act or whether to help that person in a given situation (Hayes and Scassellati, 2013). Theory of mind enables workers to quickly understand each other with a reduced amount of explicit communication.

The targets of theory of mind include not only other humans but sometimes also artifacts (Gergely et al., 1995; Schellen and Wykowska, 2019), regardless of whether they actually possess mental states similar to those of humans. This phenomenon can be utilized to facilitate natural and efficient interactions between humans and artificial agents, such as seeking human help without verbal cues (Cha and Mataric, 2016), although it may also have undesirable effects. For example, humans may make false inferences regarding what an agent is intending to do based on mere observation of its behavior. Such misunderstandings can lead to failure of collaboration or even serious accidents. In this context, autonomous artificial agents need to act with *public self-awareness*, or inference of how its behavior will be considered by its observers (Feningstein, 1975; Falewicz and Bak, 2015).

**FIGURE 1:**
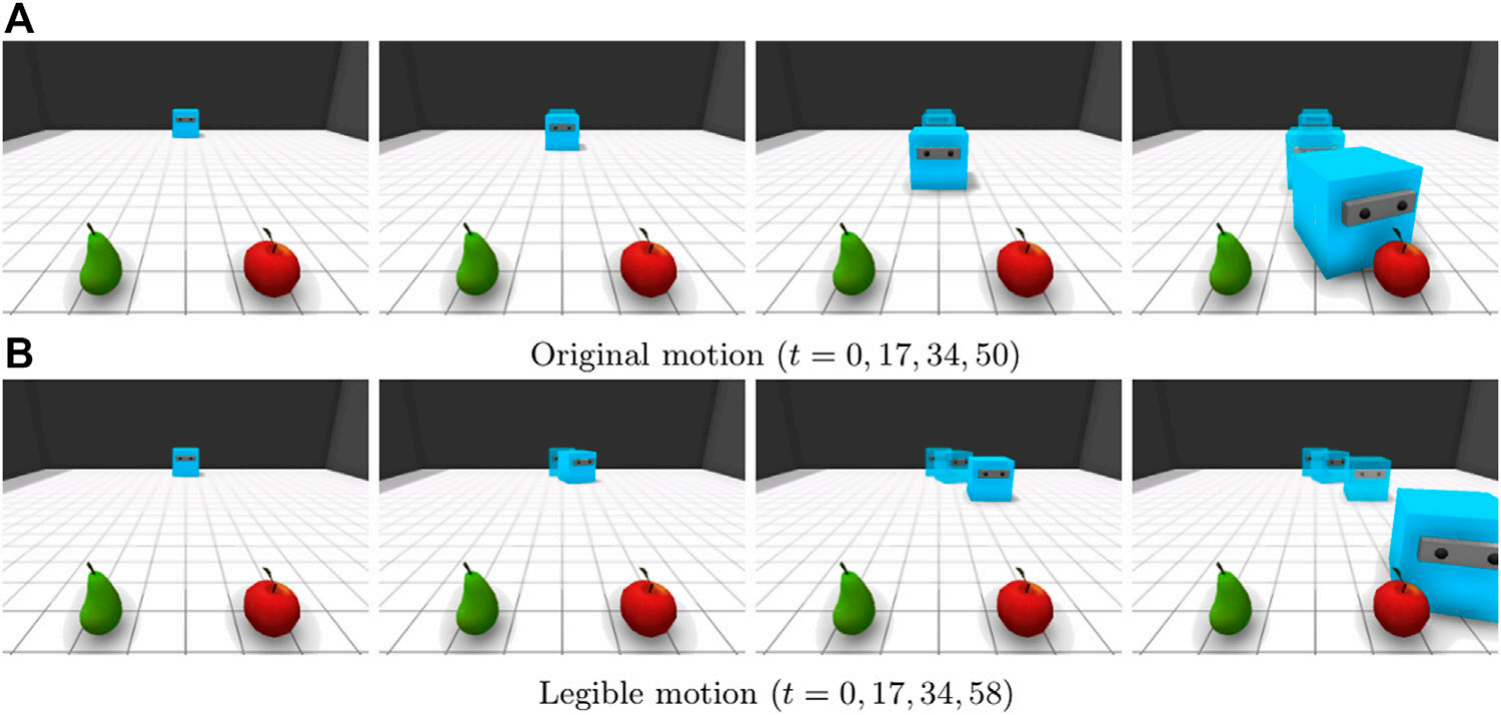
An example in which legible motion serves to improve the predictability of an agent’s intention. The blue agent is initially at the far side of the room and intends to get the apple. The agent’s movements are captured from a fixed observation point. Legible motion reduces ambiguity early on, enabling the observer to infer the true intention of the agent. **(A)** Original motion (t = 0, 17, 34, 50). **(B)** Legible motion (t = 0, 17, 34, 58).

Previous studies have proposed computational methods for enabling autonomous agents to act with awareness of an observer’s theory of mind. Dragan et al. formalized the problem of an artificial agent’s inference of the goal attributed to it by a human observer and proposed a method to generate motion that conveys a goal-directed agent’s specific intention to a human observer to either lead or mislead human inference of what the agent is aiming to do (Dragan and Srinivasa, 2014; Dragan et al., 2015b). Motion that conveys an agent’s true intention is specifically called *legible* motion. Figure 1 illustrates an example. The blue agent intends to retrieve the apple in the environment. The original motion (Figure 1A) is the result of an attempt to choose efficient motion to achieve its goal without considering its observer’s theory of mind. The agent moves straight and then turns toward the apple just in front of the observer. The observer cannot judge which fruit the agent intends to retrieve when the agent is moving straight toward the observer; thus, it is difficult for the observer to quickly predict the agent’s intention by observing the agent’s behavior. By contrast, with legible motion (Figure 1B), the agent moves toward the side corresponding to the apple from the beginning, excluding the possibility of interpretation that the agent intends to retrieve the pear. Although the time required for the agent to retrieve the apple is increased, the observer can more quickly and correctly infer that the agent intends to retrieve the apple than in the case of the original motion. Previous work on legible motion has successfully demonstrated the effectiveness of endowing an artificial agent with awareness of human theory-of-mind inference with respect to its behavior during human-robot collaboration (Dragan et al., 2015a).

**FIGURE 2:**
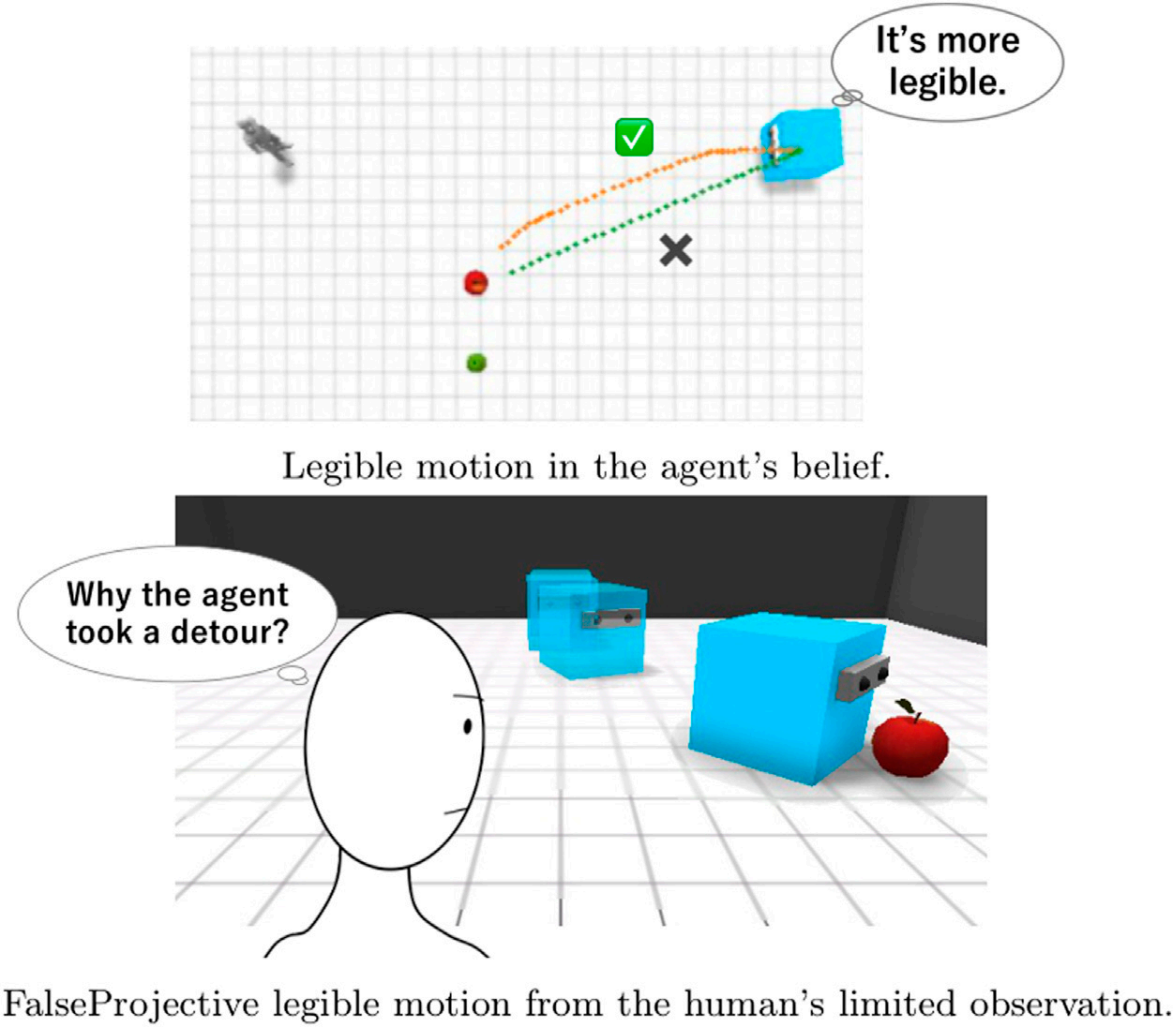
An example in which FalseProjective legible motion, which does not consider information asymmetry, fails to effectively convey the agent’s intention. FalseProjective legible motion results in showing an unuseful detour because it does not consider the fact that the human does not know where a pear is. **(A)** Legible motion in the agent’s belief. **(B)** FalseProjective legible motion from the human’s limited observation.

Previous studies assumed simple situations in which both the agent and its observer can easily share information about the environment, so they do not have to consider information asymmetry or differences in what each individual observes. Figure 2 shows a simple example in which previous method does not work due to information asymmetry. An apple and a pear are both present on the right side of the observer (Figure 2A). If the observer could observe both the apple and the pear, the motion curved to the apple could be effective because it prevented the observer from mistaking the agent’s target for the pear. However, when we see the motion from the perspective of the observer who cannot observe the pear, the early motion makes it appear as though the agent is ignoring the apple (Figure 2B). This example suggests that legible motion with the false assumption that the actor and the observer share beliefs about the environment does not always improve the legibility of an agent’s behavior and can sometimes even make it more difficult for an observer to infer the intention that the agent aims to convey.

Our main claim here is that information asymmetry is a critical factor in generating motions that convey certain intentions. To formalize this claim, we developed a novel method to generate legible motion with awareness of information asymmetry. This method is based on our previously proposed PublicSelf model, which is a computational model of public self-awareness that infers the mental states attributed to the agent by an observer (Fukuchi et al., 2018). By explicitly distinguishing the observations and beliefs of an acting agent and its observer, PublicSelf can accurately predict a human’s inference of an agent’s mind in information-asymmetric situations with partial observability, but it was not applied to generating an agent’s behavior. To validate our claim, we conducted a simulation study and a user study to compare the legible motion generated with our method, PublicSelf legible motion, with *FalseProjective* legible motion, which does not consider information asymmetry. The results showed that PublicSelf legible motion improves the predictability of an agent’s intentions compared with FalseProjective legible motion, indicating that information asymmetry is a critical problem when conveying intentions by motions and that our formalization can effectively address this problem.

This paper is structured as follows. Section 2 presents the background, explains the problem of conveying intentions by motions, and summarizes previous studies. Section 3 proposes our method of generating legible motion using PublicSelf. Section 4 describes the implementation of PublicSelf and the generation of legible motion. Section 5 reports on the two experiments conducted to evaluate PublicSelf legible motion and discusses the results. Section 6 discusses directions for future work. Section 7 concludes this paper.

## 2 Background

### 2.1 Explainability of Intelligent Agents’ Behavior in Human-Agent Collaboration

In this paper, intelligent agents (IAs) refers to an autonomous and goal-directed artificial agent that utilizes machine learning (ML) methods such as deep reinforcement learning (DRL) to achieve certain objectives. With the recent development of ML, IAs have achieved good performance in complex tasks (Mnih et al., 2015; Silver et al., 2017), and an increasing number of studies are focusing on the application of real-world robots (Kahn et al., 2018; Kalashnikov et al., 2018). Introducing ML methods can be a promising approach to realize effective goal-directed human-agent collaboration.

However, many challenges remain that hinder collaboration between people and IAs. One of the major difficulties is the lack of explainability of an agent’s future behavior. Decision-making modules that utilize modern ML tend to be a black box (Fukuchi et al., 2017; Hayes and Shah, 2017). In particular, the DRL model embeds the control logic in high-dimensional parameter space and usually does not provide human-comprehensible expressions of the agent’s plans, goals, or intentions. Therefore, most people cannot understand what an IA is aiming to do. The ability to understand and predict a human coworker’s behavior can help robots to effectively collaborate with people (Lasota and Shah, 2015; Huang and Mutlu, 2016). Similarly, human agents also should be able to better understand their coworker agents’ future behavior.

Previous studies have proposed methods to explain diverse aspects of an IA’s decision making. Saliency maps are commonly used to explain the reason a deep learning module made a specific decision based on its input modality, and they are also applied to an IA’s policy model (Tamagnini et al., 2017; Iyer et al., 2018; Mott et al., 2019). While many approaches focus on explanations for AI practitioners, Cruz et al. proposed a method for end users (Cruz et al., 2021). Their method enables an IA to explain its next action with the probability of success. Hayes et al. proposed a natural language question answering system that can handle some templates of questions about an IA’s policy such as “when do you {action}?” and “what will you do when {state}?” (Hayes and Shah, 2017).

### 2.2 Inference of IA Minds by Humans

Humans sometimes consider an artificial agent to have a mind and thus to be a valid target for theory-of-mind inference. This attitude is called the “intentional stance” (Dennett, 1987). Many studies have investigated which characteristics of an artificial agent lead people to adopt the intentional stance. Human-likeness is a factor that leads humans to adopt an intentional stance (Wimmer and Perner, 1983; Perez-Osorio and Wykowska, 2020), but even geometrical figures can be targets of the intentional stance (Gergely et al., 1995) when they appear to be goal-directed (Premack and Premack, 1997), rational (Gergely et al., 1995), self-propelled (Luo and Baillargeon, 2005), or in violation of Newtonian laws (Scholl and Tremoulet, 2000).

IAs have many of these characteristics, which suggests that people can adopt an intentional stance toward them. For example, a rational agent is expected to take actions that maximize its own utility (Jara-Ettinger et al., 2015), and the RL framework is designed to address exactly the problem of utility maximization. The utility function for a general RL agent is designed to drive that agent to achieve certain goals in an efficient (or rational) manner; positive rewards encourage the RL agent to achieve certain tasks, while negative costs urge the agent to choose more efficient actions.

### 2.3 Self-Awareness for Explainable IAs

A person can infer another person’s beliefs, goals, or intentions even without explicit communication. In effect, people can infer the minds of others simply by observing their behavior (Baker et al., 2017). However, there is also a risk of misunderstanding or of forming false beliefs about other people based on such observations. When a person adopts the intentional stance with regard to an IA and attempts to infer the agent’s mind, the same problem can arise.

In this context, objective self-awareness, or the ability of a person to recognize themselves as an object of attention (Duval and Wicklund, 1972), becomes an important component for an IA to help humans correctly understand its behavior. Objective self-awareness is considered to have two aspects: private self-awareness and public self-awareness (Feningstein, 1975; Falewicz and Bak, 2015). A privately self-aware person is self-reflective and attentive to their own thoughts. A publicly self-aware person, on the other hand, focuses on the self as a social object and is attentive to how he or she appears to others. If an IA has a private self-awareness model, it can reveal ι*, the intention that is going to be achieved by the agent. A public self-awareness model will also enable the agent to infer the intention *ι* that a human observer will attribute to it based on its behavior. On this basis, the agent can select an action *a* that will lead the observer to infer the agent’s true intention $a={\mathrm{argmax}}_{a}P(\iota =\iota *|a)$ . Similarly, an agent can also select actions that will mislead the observer to infer a particular false intention.

### 2.4 Computational Theory of Mind Model

Multiagent systems (MASs) represent one research field that aims to introduce the concept of theory of mind to artificial agents. Theory of mind ability enables agents to choose their actions based on what another agent is going to do, resulting in better performance in both cooperative and competitive situations (Zettlemoyer et al., 2009; Raileanu et al., 2018).

One of the major challenges of inferring an other’s mind is its multiply nested structure. Here, we formalize the nested inference structure using belief-desire-intention logic (Cohen and Levesque, 1990). Suppose that there are two agents, an actor agent that performs actions, and an observer agent that attempts to infer the actor’s intention based on its actions. Let us call the latter a first-order inference and denote the inferred intention *ι* ^1^:

$$\left(\text{BEL}\;observer\;\left(INTEND\;actor\;\iota^{1}\right)\right),$$

where (BEL *i X*) means agent *i* believes *X*, and (INTEND *i ι*) means agent *i* intends to achieve *ι*. Actors can also have second-order beliefs, that is, beliefs about the observer’s belief. For example,

$$\left(\text{BEL}\;actor\;\left(\text{BEL}\;observer\;\left(INTEND\;actor\;\iota^{2}\right)\right)\right)$$

means that the actor believes that “the observer believes that the actor intends to achieve *ι* ^2^.” In this paper, a superscript *k* means that the corresponding variable represents a *k*-th order belief. We can consider an arbitrary order of inference by repeating this manipulation.

Zettlemoyer et al. proposed sparse distributions over sequences (SDS) filtering (Zettlemoyer et al., 2009), an algorithm to compute the nested belief. SDS filtering can efficiently solve the problem of sequential inference about nested beliefs by utilizing a *sequence distribution*, which represents the probability distribution of an agent’s belief about the environment and the other agents’ beliefs given a set of possible sequences of states.

The computational theory-of-mind model has also been studied in the field of cognitive science. Baker et al. proposed the Bayesian theory of mind (BToM) model (Baker et al., 2017). The BToM describes an observer agent’s first-order inference of an actor agent’s mental state, such as a belief, desire, or intention, while observing the actor agent’s behavior. Equation 1 describes the inference model:

$$\begin{array}{cc} & \;P\left(b_{t+1}^{1},d^{1},\iota_{t+1}^{1}|o_{:t+1}\right)\\ \propto & \underset{\begin{array}{c} o_{t+1}^{1},s_{t+1},\\ s_{t},a_{t},b_{t}^{1},\iota_{t}^{1} \end{array}}{\sum }P\left(\iota_{t+1}^{1}|b_{t+1}^{1},d^{1},\iota_{t}^{1}\right)\cdot P\left(b_{t+1}^{1}|b_{t}^{1},o_{t+1}^{1}\right)\cdot P\left(o_{t+1}^{1}|s_{t+1}\right)\cdot P\left(o_{t+1}|s_{t+1}\right)\\ & \;\;\cdot P\left(s_{t+1}|s_{t},a_{t}\right)\cdot P\left(a_{t}|b_{t}^{1},d^{1},\iota_{t}^{1}\right)\cdot P\left(b_{t}^{1},d^{1},\iota_{t}^{1}|o_{:t}\right), \end{array}$$ (1)

where *a* is an action performed by the actor, and belief *b*_*t*_ is a probability distribution representing the probability that the environmental state is *s*_*t*_ given past observations *o*_:*t*_, i.e., *b*_*t*_(*s*_*t*_) = *P*(*s*_*t*_|*o*_:*t*_). The observer attributes mental states to the actor at time *t*, such as observation $o_{t}^{1}$ , belief $b_{t}^{1}$ , desire *d* ^1^, and intention $\iota_{t}^{1}$ based on the observer’s observation history *o*_:*t*_ from times *t* = 0, 1, …, *t*. Experiments demonstrated that BToM accurately captures human mental state judgments.

**FIGURE 3:**
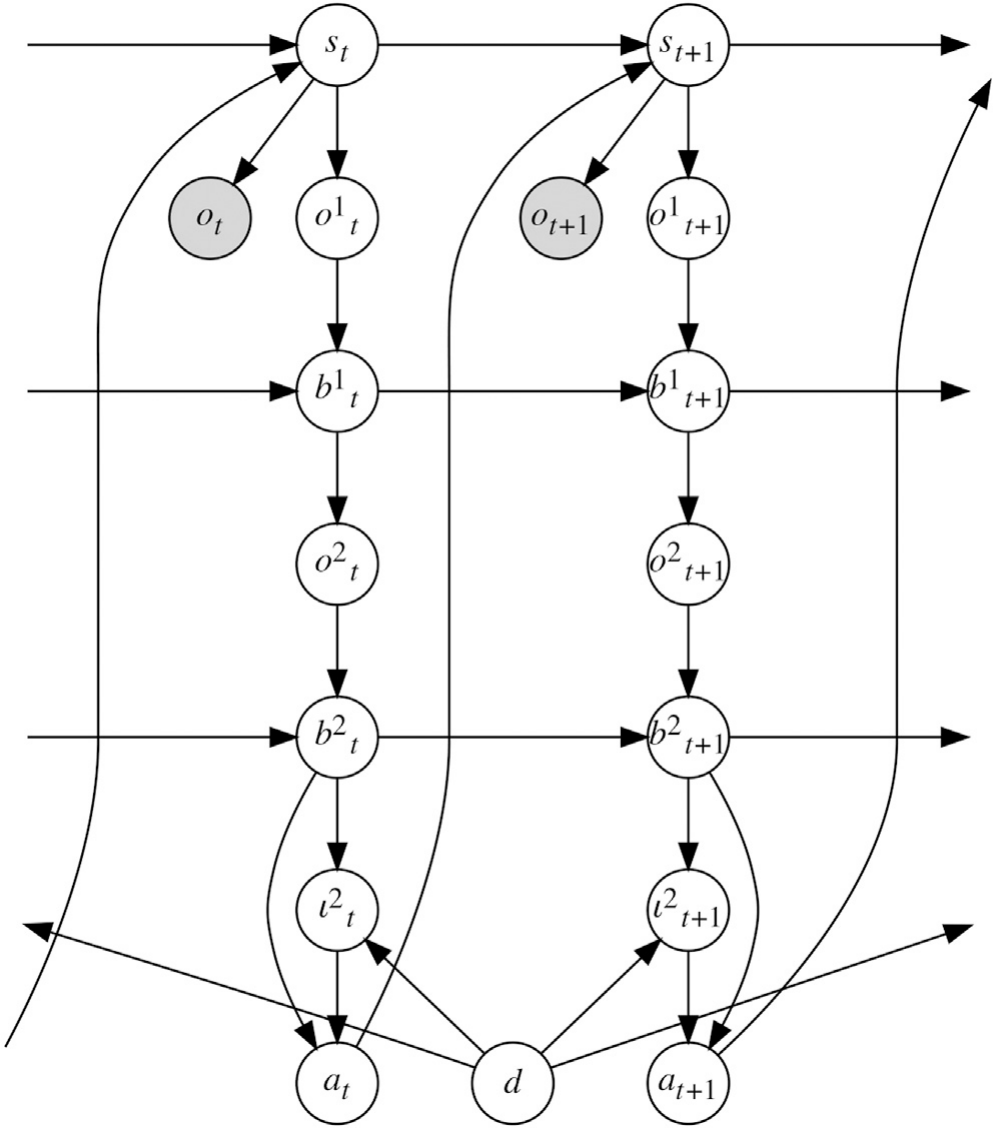
A graphical representation of PublicSelf. PublicSelf distinguishes mental states that are based on an actor agent’s actual observations, first-level beliefs of an observer’s mental state, and second-level beliefs attributed to the actor by the observer, which makes it possible to consider information asymmetry.

The PublicSelf model extended BToM to an actor’s second-order belief inference (Fukuchi et al., 2018). We can consider PublicSelf as a computational model of an actor’s public self-awareness. PublicSelf can be represented in the form of a Bayesian network (Figure 3). From the actor’s observations *o*_:*t*_, the probabilities with which belief $b_{t}^{2}$ , desire *d* ^2^, and intention $\iota_{t}^{2}$ will be attributed to the actor can be calculated:

$$\begin{array}{cc} & \;P\left(b_{t+1}^{2},d^{2},\iota_{t+1}^{2}|o_{:t+1}\right)\\ & \propto \underset{\begin{array}{c} o_{t+1}^{2},o_{t+1}^{1},b_{t+1}^{1},\\ s_{t+1},s_{t},a_{t},\\ b_{t}^{2},\iota_{t}^{2},b_{t}^{1} \end{array}}{\sum }P\left(\iota_{t+1}^{2}|b_{t+1}^{2},d^{2},\iota_{t}^{2}\right)\cdot P\left(b_{t+1}^{2}|b_{t}^{2},o_{t+1}^{2}\right)\cdot P\left(o_{t+1}^{2}|b_{t+1}^{1}\right)\cdot P\left(b_{t+1}^{1}|b_{t}^{1},o_{t+1}^{1}\right)\\ & \;\cdot P\left(o_{t+1}^{1}|s_{t+1}\right)\cdot P\left(o_{t+1}|s_{t+1}\right)\cdot P\left(s_{t+1}|s_{t},a_{t}\right)\cdot P\left(a_{t}|b_{t}^{2},d^{2},\iota_{t}^{2}\right)\cdot P\left(b_{t}^{2},d^{2},\iota_{t}^{2}|o_{:t}\right). \end{array}$$ (2)

In PublicSelf, first, a belief about the environment is constructed based on the actor’s own visual observations. That is, an observation of an object *o*_*t*_ increases the likelihood of the actor’s belief of possible environment states *s* in which the object exists at the observed position. Then, the observer’s belief about the environment, denoted by *b* ^1^, is considered, i.e., $b_{t}^{1}\left(s_{t}\right)=P\left(s_{t}|o_{:t}^{1}\right)$ , where *o* ^1^ is an inference concerning the observer’s observations. From *b* ^1^, one can then estimate *o* ^2^ and *b* ^2^, the observation and belief attributed to the actor by the observer, where $b_{t}^{2}\left(s_{t}\right)=P\left(s_{t}|o_{t}^{2}\right)$ .

The important point of PublicSelf is that it distinguishes mental states that are based on an actor agent’s actual observations, first-level beliefs of an observer’s mental state, and second-level beliefs attributed to the actor by the observer. This ability allows PublicSelf to infer the intention the observer attributes to the actor while considering information asymmetry. A user study demonstrated that PublicSelf enables an IA to accurately infer the mental state attributed to it by a human observer in situations where partial observability causes information asymmetry between an actor and its observer. However, previous work on PublicSelf focused only on the accuracy with which it infers the mental states attributed to an IA by a human observer, and PublicSelf has not previously been applied to generating an agent’s actions based on the inference.

### 2.5 Generating Motions That Convey Intentions

Dragan et al. proposed a method for generating an artificial agent’s behavior with awareness of a human observer’s theory of mind, specifically, behaviors that communicate the agent’s intention to a human observer. In particular, *legible motion* aims to allow an observer to quickly and correctly infer an agent’s intention (Dragan et al., 2015a). By means of legible motion, an agent attempts to increase the probability that the intention an observer attributes to it will match its true intention.

In previous studies on the generation of legible motion, it was assumed that the environment is limited and that both the human observer and the artificial agent share complete information about the environment, such as what exists and where it exists. However, most actual collaboration scenarios are subject to uncertainty, and information asymmetry typically exists between human and artificial agents, meaning that one agent may possess information that the other does not. Different observations result in different beliefs, which is important to consider when modeling human theory of mind. Information asymmetry is deliberately employed in daily social acts including deception, and many related psychological experiments, such as false-belief tasks, have been performed (Wimmer and Perner, 1983). Therefore, this paper claims that an artificial agent needs to handle information asymmetry between the agent and a human observer when generating publicly self-aware behavior.

To validate our claim, we compare PublicSelf legible motion that considers information asymmetry with legible motion that does not account for information asymmetry, which we call *FalseProjective*.

With FalseProjective, an actor does not distinguish its own belief regarding the environment from an observer’s belief and falsely identifies its own belief with the observer’s one.

The work by Nikolaidis et al. is the most closely related to this paper’s concept (Nikolaidis et al., 2016). They proposed a method for generating legible motion considering the effect of a human observer’s viewpoint. However, their focus was on depth uncertainty and occlusion of a robot arm and did not target the differences in beliefs about the world state such as what is where in the environment.

## 3 Conveying Intentions by Motions With Awareness of Information Asymmetry

The claim of this paper is that we need to consider information asymmetry to generate motions that convey a certain intention to other agents. To validate this claim, we develop a method for generating such motions by extending the PublicSelf model and compare the generated motions with those that do not consider information asymmetry in an approach similar to that in previous work.

**Algorithm 1:** Generating PublicSelf legible motion.

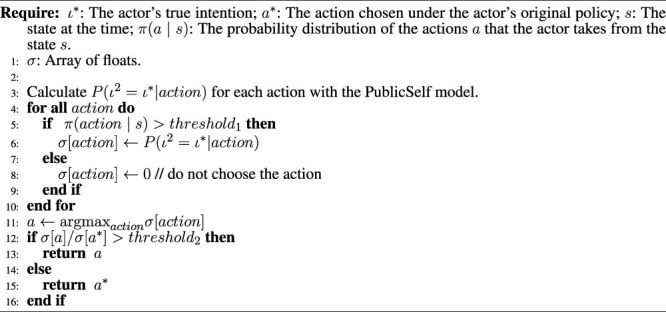

**Algorithm 2:** Updating the PublicSelf model.

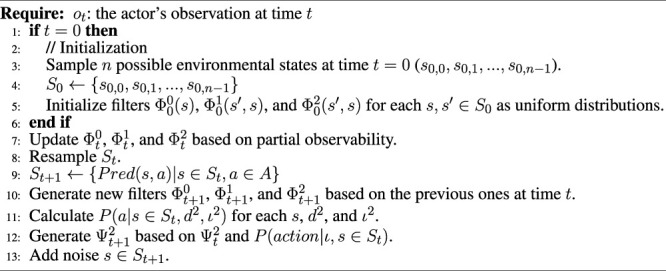

A brief description of our method is as follows: By extracting specific probabilities under the conditions of each action *a*_*t*_ in the summation of Eq. 2, we can calculate *P*(*b* ^2^, *d* ^2^, *ι* ^2^|*o*, *a*), the probability that a human observer will attribute mental states (*b* ^2^, *d* ^2^, *ι* ^2^) to an actor given that actor’s specific action *a*. On this basis, we can select the action that will most effectively express the actor’s mental state:

$$a={\mathrm{argmax}}_{a}P\left(b^{2},d^{2},\iota^{2}|o,a\right).$$ (3)

In particular, by marginalizing over *b* ^2^ and *d* ^2^, we can obtain the action that will most effectively express the agent’s intention, or the most legible action. Because PublicSelf captures the differences in observations (*o*, *o* ^1^, *o* ^2^) and beliefs (*b*, *b* ^1^, *b* ^2^) between an actor and an observer agent, we can generate motions while considering information asymmetry.

Algorithm 1 presents the procedure for generating legible motion with PublicSelf. Here, *π*(*a*∣*s*) is a probability distribution of taking the actions *a* given a state *s*, which corresponds to a typical formulation of an actor’s decision-making model in reinforcement learning. We do not simply choose an action based on Eq. 3. In other words, we do not adopt actions that are unlikely to be chosen under the actor’s original policy. Instead, we calculate the increase in the probability that an observer will infer the actor’s true intention that is achieved by changing the actor’s original action to the action identified by PublicSelf as the most legible; we do not change the actor’s action if the legible action does not increase the probability sufficiently to balance the cost of taking that legible action. In the situation presented in Figure 1, for example, the actor would perform quite a wide turn and take a long time to achieve the original goal if all actions were selected strictly in accordance with Eq. 3.

## 4 Fetchfruit Task and Implementation

### 4.1 Environment of the FetchFruit Task

We developed the FetchFruit task in a simulated environment (Figures 1, 2) and implemented our method of generating legible motions for an IA in this simulated environment. The environment is a square room containing an apple, a pear, an actor, and a human observer. The initial positions of the actor and the observer are fixed.

The actor’s actions are driven by a policy for the retrieval of an apple or a pear. Our method is independent of the implementation of the policy model as long as the probability of the actor taking each action can be calculated. In this paper, we use the asynchronous advantage actor-critic (A3C) algorithm (Mnih et al., 2016), which is one of the most representative algorithms for DRL. Every 0.5 s, the actor selects an action from the action space *A*, which is composed of three discrete actions: accelerate forward, turn clockwise, and turn counterclockwise.

The environmental state *s*_*t*_ is composed of the locations of the apple and pear, the area that is within the observer’s sight, and the actor’s state, which includes the actor’s location, velocity, and direction as well as the area within the actor’s sight. In this paper, only the actor’s state changes over time.

The human observer does not move and observes the environment from a fixed viewpoint. The observer can acquire only information that is in his/her field of view. That is, the observer can know where a fruit is only if s/he can see it. Similarly, the actor’s location, velocity, and direction are provided when the observer can see the actor, but none of the above is provided when the actor is out of the observer’s field of view.

### 4.2 PublicSelf Model

Algorithm 2 shows how Eq. 2 is calculated in our implementation. There are infinite possible environmental states because *s*_*t*_ consists of continuous values and is subject to uncertainty due to partial observability. We solve the problem by sampling possible states to simplify the state space, which is analogous to the sequential Monte Carlo method (Doucet et al., 2001). We first randomly sample *n* states *s*_0,0_, *s*_0,1_, …, *s*_0,*n*−1_ from among the possible states. The implementation of PublicSelf is based on the SDS filtering concept (see Section 2.4). PublicSelf includes four filters for inferring the beliefs and desires that the observer will attribute to the actor:

$$\begin{array}{ccc} \Phi_{t}^{0}\left(s_{:t}\right) & \propto & b^{0}\left(s_{:t}\right),\\ \Phi_{t}^{1}\left(s_{:t}^{\prime },s_{:t}\right) & \propto & \underset{b_{t}^{1}}{\sum }b_{t}^{1}\left(s_{:t}^{\prime }\right)\cdot \delta \left(b_{t}^{1},B^{obs}\left(s_{:t}\right)\right),\\ \Phi_{t}^{2}\left(s_{:t}^{\prime },s_{:t}\right) & \propto & \underset{b_{t}^{2}}{\sum }b_{t}^{2}\left(s_{:t}^{\prime }\right)\cdot \delta \left(b_{t}^{2},B^{act}\left(s_{:t}\right)\right),\\ \Psi_{t}^{2}\left(d^{2},s_{:t}\right) & \propto & P\left(d^{2}|s_{:t}\right). \end{array}$$

The delta function *δ*(*α*, *β*) returns a value of 1 when *α* = *β* and a value of 0 otherwise. *B* ^*obs*^(*s*_:*t*_) and *B* ^*act*^(*s*_:*t*_) return the observer’s and actor’s beliefs, respectively, given the history of the environmental states. Here, for simplicity, we will suppose that the transitioning of the environmental state is a Markov process and *s*_:*t*_ can be denoted as *s*_*t*_. $\Phi_{t}^{0}\left(s_{t}\right)$ denotes the actor’s belief regarding the environment. $\Phi_{t}^{1}\left(s_{t}^{\prime },s_{t}\right)$ is the probability that the observer will believe that the environmental state is $s_{t}^{\prime }$ when the actual environmental state is *s*_*t*_. $\Phi_{t}^{2}\left(s_{t}^{\prime },s_{t}\right)$ is the probability that the observer will infer that the actor believes that the environmental state is $s_{t}^{\prime }$ when the actual state is *s*_*t*_. $\Psi_{t}^{2}\left(d^{2},s_{t}\right)$ represents the probability with which desire *d* ^2^ will be attributed to the actor by the observer given state *s*_*t*_. These filters are initialized as uniform distributions.

Based on the actor’s observation *o*_*t*_, $\Phi_{t}^{0}\left(s_{t}\right)$ is updated by multiplying it by the observation probability *P*(*o*_*t*_|*s*_*t*_) because *P*(*s*_*t*_|*o*_*t*_) ∝ *P*(*s*_*t*_|*o*_*t*−1_) ⋅ *P*(*o*_*t*_|*s*_*t*_). We can similarly update $\Phi_{t}^{1}\left(s_{t}^{\prime },s_{t}\right)$ and $\Phi_{t}^{2}\left(s_{t}^{\prime },s_{t}\right)$ by estimating the actor’s and observer’s observations *o* ^1^ and *o* ^2^ under each *s*_*t*_ and multiplying them by the observation probabilities $P\left(o_{t}^{1}|s_{t}^{\prime }\right)$ and $P\left(o_{t}^{2}|s_{t}^{\prime }\right)$ :

$$\begin{array}{ll} \Phi_{t}^{0}\left(s_{t}\right) & \leftarrow \Phi_{t}^{0}\left(s_{t}\right)\cdot P\left(o_{t}|s_{t}\right),\\ \Phi_{t}^{1}\left(s_{t}^{\prime },s_{t}\right) & \leftarrow \Phi_{t}^{1}\left(s_{t}^{\prime },s_{t}\right)\cdot \underset{o_{t}^{1}}{\sum }P\left(o_{t}^{1}|s_{t}^{\prime }\right)\cdot \delta \left(o_{t}^{1},O^{obs}\left(s_{t}\right)\right),\\ \Phi_{t}^{2}\left(s_{t}^{\prime },s_{t}\right) & \leftarrow \Phi_{t}^{2}\left(s_{t}^{\prime },s_{t}\right)\cdot \underset{o_{t}^{2}}{\sum }P\left(o_{t}^{2}|s_{t}^{\prime }\right)\cdot \delta \left(o_{t}^{2},O^{act}\left(s_{t}\right)\right), \end{array}$$

where *O* ^*obs*^(*s*_*t*_) and *O* ^*act*^(*s*_*t*_) return the observer’s and actor’s observations, respectively, under *s*_*t*_.

During the update process, an *s* ∈ *S*_*t*_ may appear such that $\sum_{b^{2}}b^{2}\left(s\right)=0$ , which means that the observer no longer thinks that the actor holds any belief about *s* at all. We resample *S*_*t*_ by removing such states *s* with zero probability and making branches of samples with high probability.

The actor’s state branches depending on the actor’s choice of action. Let $S_{t+1}^{a_{t}}$ be a set of predicted environmental states to which the actor’s action *a*_*t*_ will lead from *s* ∈ *S*_*t*_, and let *S*_*t*+1_ be the union of the states predicted under each action, ${⋃}_{a}S_{t+1}^{a}$ . The function *Pred* : *S*_*t*_ × *A* → *S*_*t*+1_ returns the states at time *t* + 1 to which the environment transitions with each action from *S*_*t*_. In this study, we trained a model for *Pred* by means of supervised learning. The new values of the filters Φ_*t*+1_ are inherited from the previous values:

$$\begin{array}{ll} \Phi_{t+1}^{0}\left(Pred\left(s_{t},a\right)\right) & \leftarrow \Phi_{t}^{0}\left(s_{t}\right),\\ \Phi_{t+1}^{1}\left(Pred\left(s_{t}^{\prime },a^{\prime }\right),Pred\left(s_{t},a\right)\right) & \leftarrow \Phi_{t}^{1}\left(s_{t}^{\prime },s_{t}\right)\cdot \delta \left(a^{\prime },a\right),\\ \Phi_{t+1}^{2}\left(Pred\left(s_{t}^{\prime },a^{\prime }\right),Pred\left(s_{t},a\right)\right) & \leftarrow \Phi_{t}^{2}\left(s_{t}^{\prime },s_{t}\right)\cdot \delta \left(a^{\prime },a\right). \end{array}$$

We assume that the actor can have only two intentions, namely, *ι*_*a*_ and *ι*_*p*_, which are the intention to retrieve an apple and that to retrieve a pear, respectively. We also consider that the desire to retrieve a fruit directly generates the intention to retrieve it, that is, $P\left(\iota^{2}=\iota_{a}|d^{2}=d_{a}\right)=P\left(\iota^{2}=\iota_{p}|d^{2}=d_{p}^{2}\right)=1$ , where *d*_*a*_ and *d*_*p*_ are the utility functions when the actor’s target is an apple and a pear, respectively. Then, $P\left(a|s^{2},d_{a}^{2},\iota_{a}^{2}\right)$ simplifies to $P\left(a|s^{2},d_{a}^{2}\right)$ , which can be estimated with a model-free RL algorithm.

$$\Psi_{t+1}^{2}\left(d^{2},Pred\left(s_{t},a\right)\right)\leftarrow \Psi_{t}^{2}\left(s_{t}\right)\cdot P\left(a|s_{t},d^{2}\right)$$

To generate legible motion, we need to determine *P*(*ι* ^2^|*a*), the probability that the observer will attribute an intention *ι* ^2^ to the actor given action *a*. In our implementation, we equate *ι* ^2^ with *d* ^2^ and calculate *P*(*d* ^2^|*a*) instead of *P*(*ι* ^2^|*a*). *P*(*d* ^2^|*a*) can be obtained from the four filters as follows:

$$\begin{array}{ll} & P\left(d^{2}|a\right)\\ = & \underset{s\in S_{t+1}}{\sum }\Psi^{2}\left(s,d^{2}\right)\underset{s^{\prime }\in S_{t+1}}{\sum }\Phi^{2}\left(s^{\prime },s\right)\underset{s^{\prime \prime }\in S_{t+1}^{a}}{\sum }\Phi^{1}\left(s^{\prime \prime },s^{\prime }\right)\cdot \Phi^{0}\left(s^{\prime \prime }\right). \end{array}$$

### 4.3 Generating FalseProjective Legible Motion

Since the PublicSelf model infers the actor’s own belief *b* ^0^, PublicSelf can also be used to generate FalseProjective legible motion. A filter $\Psi_{t}^{0}\left(s,d^{0}\right)$ , which represents the probability of an actor’s own desire *d* ^0^ estimated independently of the observer, enables us to generate FalseProjective legible motion in a manner similar to the generation of PublicSelf legible motion.

### 4.4 Generated Motion

Here, we present the motion generated in the example FetchFruit scenarios. We consider five example scenarios: Center, Side-Visible, Side-Invisible, Blind-Inside, and Blind-Outside. Table ?? summarizes the settings for these scenarios, which cover all 2 × 2 possibilities with regard to whether the observer can see the actor’s target and/or the nontarget objects. In every scenario, the target and nontarget objects are adjacent to each other. The actor is assumed to observe the positions of both fruits from its initial position; consequently, the actor does not need to explore the environment but simply moves to its target. We generated three types of motion in each of the example scenarios: the original motion, the FalseProjective legible motion, and the PublicSelf legible motion.

**FIGURE 4:**
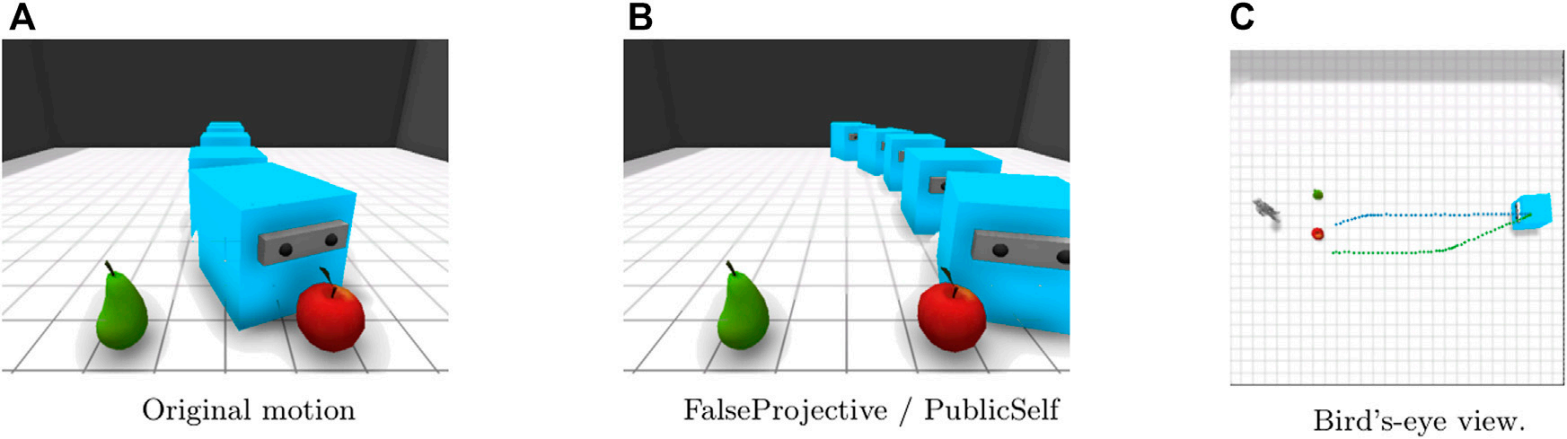
Motion in the Center scenario. Blue: Original. Orange: FalseProjective. Green: PublicSelf. FalseProjective and PublicSelf let the observer correctly infer the actor’s target by showing the curved movement from the beginning. **(A)**Original motion. **(B)**FalseProjective/PublicSelf. **(C)**Bird’s-eye view.

In the Center scenario (Figure 4), the apple and pear are immediately in front of the observer. It is difficult to quickly infer the actor’s intention from the original motion because the actor first moves straight to the point between the apple and the pear (Figure 4A), whereas the FalseProjective motion enables the observer to infer the actor’s intention more quickly because of the agent’s curved movement toward its actual target (Figure 4B). The PublicSelf legible motion follows the same route as the FalseProjective motion.

**FIGURE 5:**
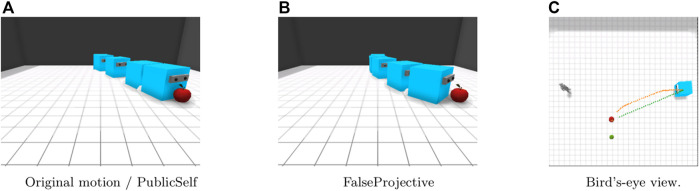
Motion in the Side-Visible scenario. FalseProjective shows a curved movement to avoid being considered that the actor intends to retrieve the pear, but is less effective than the straightforward movement of original and PublicSelf due to the observer’s limited view. **(A)**Original motion/PublicSelf. **(B)**FalseProjective. **(C)**Bird’s-eye view.

In the Side-Visible scenario (Figure 5), the human observer can observe only the apple. With the original motion trajectory, the actor moves directly to the apple. Because the observer cannot see the pear next to the apple, the original motion presents much less ambiguity than in the Center scenario. Here, PublicSelf generates the same trajectory as that for the original motion. The FalseProjective motion, on the other hand, follows a different trajectory; the actor first moves forward toward the observer and then follows a curved trajectory toward the apple. This motion would avoid ambiguity if the observer knew the location of the pear; however, due to the observer’s limited view, this motion may instead lead the observer to believe that the actor’s target is behind him or her; thus, the actor’s intention is less clear in this case than it would be if the actor moved directly to the apple.

**FIGURE 6:**
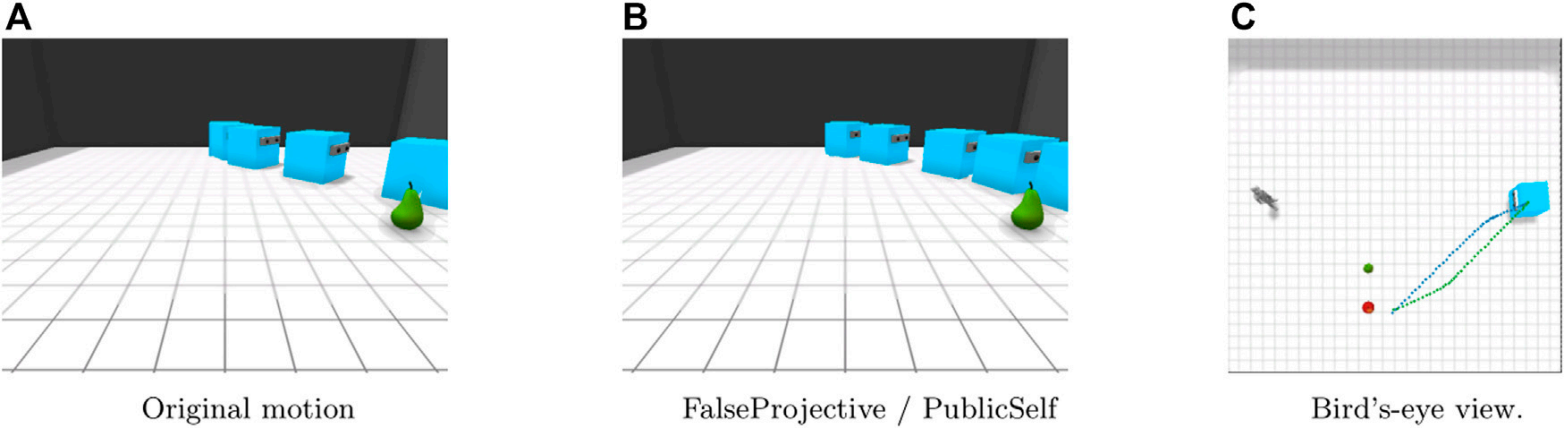
Motion in the Side-Invisible scenario. FalseProjective and PublicSelf motion trajectories successfully avoid misleading the observer into considering that the actor is moving toward the pear. **(A)**Original motion. **(B)**FalseProjective/PublicSelf. **(C)**Bird’s-eye view.

Figure 6 shows the results in the Side-invisible scenario. With the original motion trajectory, the actor first begins to move to the space between the apple and pear and then shows a gently curved motion. Early motion could mislead the observer into thinking that the actor intends to retrieve the pear, whereas through the PublicSelf legible motion, the actor can convey that the pear is less likely to be its target by making a detour to avoid causing the observer to misunderstand the actor’s intention. Here, the PublicSelf legible motion is the same as the FalseProjective motion.

**FIGURE 7:**
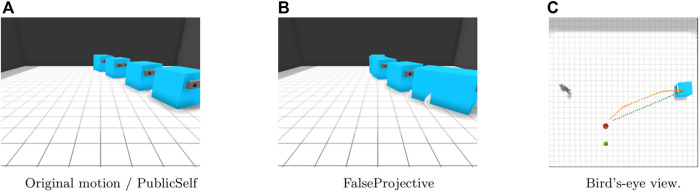
Motion in the Blind-Inside scenario. Because the observer cannot see any target candidates, the detour of FalseProjective provides no information about the actor’s target. **(A)**Original motion/PublicSelf. **(B)**FalseProjective. **(C)**Bird’s-eye view.

**FIGURE 8:**
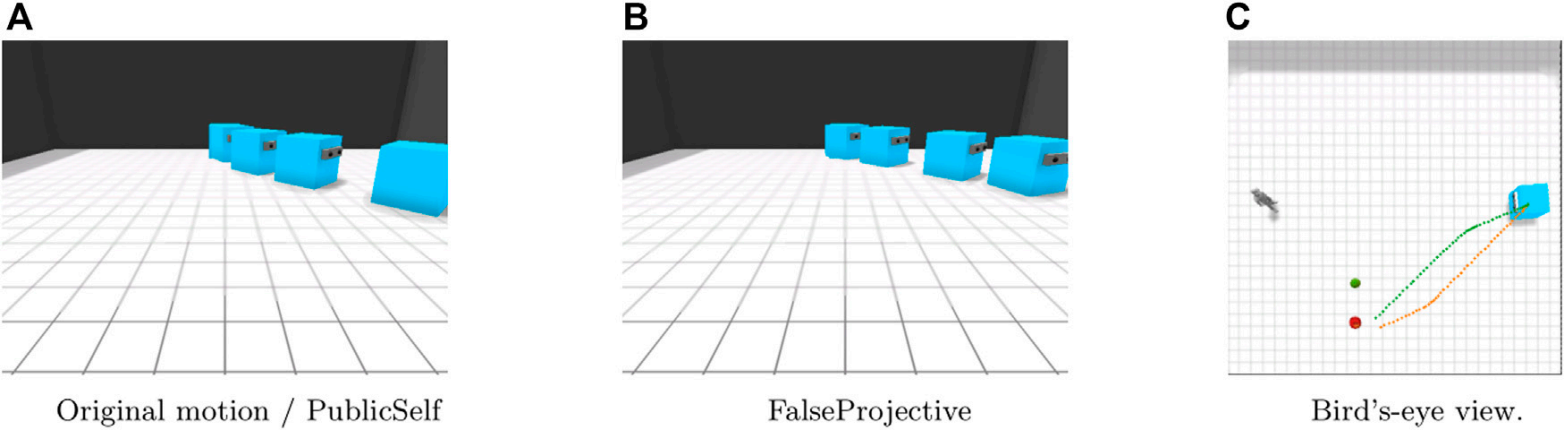
Motion in the Blind-Outside scenario. **(A)**Original motion/PublicSelf. **(B)**FalseProjective. **(C)**Bird’s-eye view.

In the two Blind scenarios (Figures 7, 8), the actor’s motion does not convey any information to the observer, who does not know the location of either the apple or the pear; therefore, the FalseProjective motion merely introduces a detour and increases the time required to retrieve the apple. By contrast, the PublicSelf legible motion does not change the actor’s actions because PublicSelf can infer that changing the motion would have no effect on the observer’s inference.

## 5 Experiments

### 5.1 Simulation Study

#### 5.1.1 Overview

The simulation study aimed to investigate the scalability of PublicSelf legible motions. We prepared additional FetchFruit scenarios beyond the examples in Section 4.4, and compared the legibility of the three motion types using artificial observers that were trained to classify an actor’s intentions from observations of its motion. We also analyzed the effects of information asymmetry on generating legible motions to investigate whether PublicSelf legible motion could effectively handle information asymmetry.

#### 5.1.2 Procedure

We built two FetchFruit datasets for 1) training the artificial observers and 2) evaluating PublicSelf legible motion. Both are composed of the captured motions of an actor agent from the observer’s viewpoint and ground-truth labels of the object that the actor intended to retrieve. An apple and a pear were randomly spawned within the field of view of the actor agent, but they were not necessarily within the observer’s field of view. The datasets do not include conditions in which both an apple and a pear are in the observer’s field of view because an observer cannot distinguish an actor’s intention in such conditions. We prepared all three motion patterns for each fruit’s position conditions. For the training datasets, we used only trials in which all three types of motions were completely identical to eliminate bias with regard to the motion type; 1,847 conditions satisfied this requirement. For the evaluation of PublicSelf, we excluded conditions in which the three motion patterns were all identical to focus on the differences between the motions. We acquired 695 conditions for the evaluation dataset.

The artificial observers were deep-learning classification models that were composed of convolutional layers, a long short-term memory layer, and fully connected layers. They were trained to infer whether the actor’s target was the apple or pear based on sequences of captured images as a supervised learning problem using the training dataset.

We used the average probability of the inference of five classification models as the score for the legibility of each trajectory. The interrater reliability of the classification models was 0.936 (ICC(3, *k*)).

Depending on the trajectories, the lengths of different trials could be different even when the initial positions were the same. Therefore, to compare different motion types under the same condition, we aligned the lengths of all trials with the same initial position by truncating them at the time when the shortest trial ended.

#### 5.1.3 Hypotheses

We expected that PublicSelf legible motions could adaptively handle various scenarios and thus the artificial observers would be able to infer the actor’s intentions more accurately from PublicSelf than from other motion types:

**H1** Legibility scores of PublicSelf are higher than those of the other motions.

In particular, we expected that PublicSelf would show better performance than FalseProjective in situations with information asymmetry but not in symmetric situations if the differences in observations and beliefs between the actor and the observer are a key factor for generating legible motion in situations with information asymmetry, and PublicSelf could successfully capture it.

**H2** Legibility scores of PublicSelf are higher than those of FalseProjective in situations *with* information asymmetry but show no differences in situations *without* information asymmetry.

Here, in the FetchFruit task, information asymmetry refers to the situation in which the observer does not know the locations of both an apple and a pear while the actor does.

#### 5.1.4 Results

**FIGURE 9:**
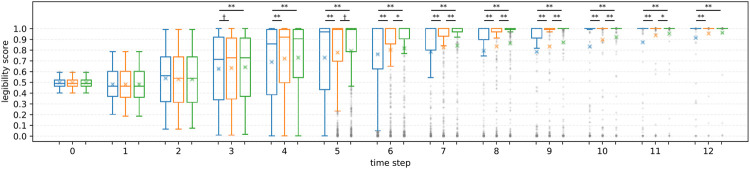
Changes in scores for each motion. Left: Original, Center: FalseProjective, Right: PublicSelf. X marks indicate the mean values. The symbols represent the results of multiple paired t-tests (**: *p* < 0.01, *: *p* < 0.05, †: *p* < 0.1). On average, PublicSelf scored higher than the other motions.

**R1** Figure 9 shows the averaged legibility scores for each motion type. Overall, PublicSelf legible motion showed higher scores than the other motions (3 ≤ *t*) except for time step *t* = 1, 2, where original motion scored the highest. For statistical analysis, we conducted Friedman test to compare the motion types for each time step. Because multiple testing inflates the type I error rate, *p* values were adjusted with the Holm-Sidak method. The results showed statistical significances in motion type at 3 ≤ *t* ≤ 12 (*p* = 0.036 at *t* = 3 and *p* < 0.01 at 4 ≤ *t*). As post hoc analysis, we conducted multiple comparisons among the three motions for each time step using Wilcoxon signed-rank tests with the Holm-Sidak adjustments. Figure 9 shows the results of the post hoc comparisons. Both FalseProjective and PublicSelf recorded significantly higher scores than the original motion at 4 ≤ *t* ≤ 12, and the scores of PublicSelf were significantly higher than those of FalseProjective at 6 ≤ *t* ≤ 10 and marginally significant at *t* = 5. The maximum effect sizes of the Wilcoxon signed-rank test *r* was 0.23 at *t* = 12 between original and FalseProjective, 0.29 at *t* = 12 between original and PublicSelf, and 0.17 at *t* = 9 between FalseProjective and PublicSelf. From these results, we considered that we can accept **H1**.

**FIGURE 10:**
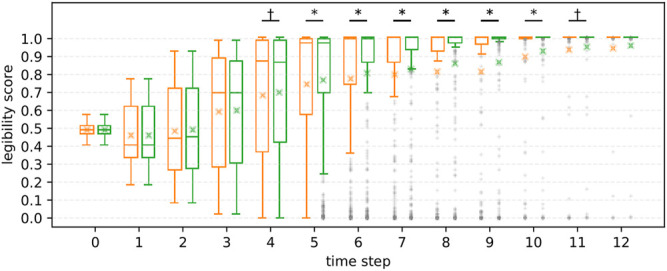
Legibility scores of FalseProjective and PublicSelf in situations *with* information asymmetry. Left: FalseProjective, Right: PublicSelf.

**FIGURE 11:**
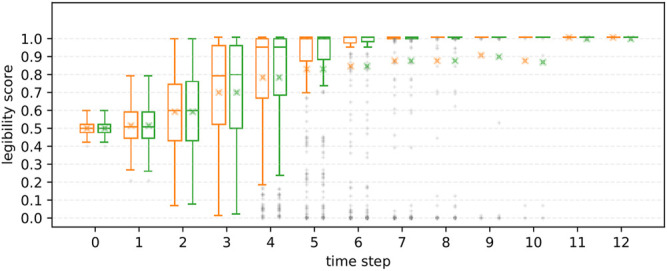
Legibility scores of FalseProjective and PublicSelf in situations *without* information asymmetry. No significant difference was found between FalseProjective and PublicSelf.

**R2** We further investigated the effect of information asymmetry on the results. Figures 10, 11 illustrate the differences in scores between FalseProjective and PublicSelf in situations with and without information asymmetry. In situations with information asymmetry, PublicSelf recorded better scores than FalseProjective, and Wilcoxon signed-rank tests revealed that there were significant differences in 5 ≤ *t* ≤ 10. However, there was little difference in situations without information asymmetry. We found no significant differences between the two motions. These results support our hypothesis **H2**.

#### 5.1.5 Summary of Simulation Study

The first results (**R1**) demonstrated that PublicSelf scored higher than the other two motions for the large dataset. This result supports **H1** and suggests that PublicSelf legible motion could work robustly even in scenarios other than the examples. In addition, the second results (**R2**) supported **H2**. We found significant improvements from PublicSelf compared to FalseProjective in situations with information asymmetry but did not in situations without information asymmetry. From this result, we conclude that information asymmetry is a critical factor when conveying intentions with motions and that we can successfully address it by explicitly introducing the differences between the observations and beliefs of the actor and those of the observer to the model.

### 5.2 User Study

#### 5.2.1 Overview

In a user study, we investigated human inference of an actor agent’s mind against the three motion types to verify that PublicSelf legible motion is effective for human observers. We compared the accuracy of the inferences among the three motion types. We also looked into the participants’ psychological perceptions of each motion type with a simple questionnaire.

#### 5.2.2 Procedure

Twelve undergraduate and graduate students (6 female and 6 male; aged 20–24, *M* = 22.6, SD = 1.83) were recruited with compensation of 750 JPY and asked to predict whether an actor would reach an apple or a pear while observing its movement. A user interface displayed the actor’s motion at ten frames per second, and the participants observed the actor and pushed the F key or the J key to, respectively, indicate whether they believed that the actor intended to retrieve the apple or the pear. The participants could also express that they could not determine which fruit the actor intended to retrieve by pushing neither key. The correspondence between the keys and the answers was randomly chosen for each participant.

Before the experiment, we instructed the participants that there would always be one apple and one pear at random locations in the room, while the initial positions of the observer and actor would be fixed. We also told them that the goal of the actor would be determined randomly for each scenario and that the actor might intend to retrieve either fruit.

After familiarization, the participants were presented with nine scenarios for each motion type: original, FalseProjective, and PublicSelf. The order effect was fully counterbalanced. These nine scenarios included the example scenarios presented in Figures 4–8, and the other scenarios were fake scenarios in which the locations of the apple and pear were randomized. We included these fake scenarios to decrease the possibility that the participants would notice that they were being presented with the same scenarios for each motion type.

We collected subjective measures by means of a simple questionnaire after the inference session by asking three Likert-scale questions:

Q1. It was easy to predict which fruit the agent was going to retrieve (Legibility 1).

Q2. The agent moved in a manner that made its intention clear (Legibility 2).

Q3. The agent’s behavior was consistent (Consistency).

Q1 and Q2 were questions adopted in a previous legible motion study (Dragan et al., 2015a) to ask participants whether they thought the actor’s motion was legible. We added the question about the consistency of the agent’s behavior to investigate the observers’ perceptions of the unique behavior of FalseProjective legible motions in Side-Visible because we hypothesized that FalseProjective’s roundabout behavior would be perceived as inconstant from the observer’s perspective.

#### 5.2.3 Hypotheses

We investigated the results for the human inferences using two metrics: rapidity and accuracy. Higher rapidity means that the motion presents less ambiguity, thus the participants required less time to infer the actor’s true intention. Higher accuracy means that the motion resulted in fewer incorrect inferences.

Tables 1, 2 summarize our hypotheses on human inferences. In the Center scenario, we hypothesized that FalseProjective and PublicSelf legible motions will result in better performance in the rapidity metrics because they present much less ambiguity than original motion does. On the other hand, we felt that there would be no difference in the accuracy metrics because none of the motions would mislead observers to make wrong inferences. In the Side-Visible scenario, we considered that FalseProjective, which acted with extra consideration of the actor and the non-target object, would be less rapid than the original and PublicSelf legible motions, which showed straightforward movements to the actor’s target object. In addition, we expected that FalseProjective motion’s early movement would mislead the observers to think that the actor was ignoring the target object and therefore would result in lower accuracy. A similar hypothesis about the accuracy metrics was formulated for Side-Invisible, in which the original motion could lead to incorrect inferences. We also considered that FalseProjective and PublicSelf legible motion would enable observers to rapidly infer the actor’s correct intention by exaggerating that the object in the actor’s view was not the target.

**TABLE 1:** Hypothses on the *rapidity* measure.

	Original	FalseProjective	PublicSelf
Center	✗	✓	✓
Side-Visible	✓	✗	✓
Side-Invisible	✗	✓	✓

**TABLE 2:** Hypothses on the *accuracy* measure.

	Original	FalseProjective	PublicSelf
Center	✓	✓	✓
Side-Visible	✓	✗	✓
Side-Invisible	✗	✓	✓

In terms of the subjective metrics, we expected that the PublicSelf legible motion would earn the best legibility scores among the three motion types. We assumed that original motion would earn the worst legibility because of its ambiguous nature. The FalseProjective legible motion was expected to be better than the original motion from the perspective of ambiguity but worse than the PublicSelf legible motion due to the unnecessary detours in the Side-Invisible and Blind-Inside scenarios. We also considered that FalseProjective legible motion would earn worse consistency scores than the others because the detours that mislead participants for wrong inferences could be perceived as inconstant from the observer’s perspective. We did not expect that participants would sense inconsistency in original motion because it provided only ambiguity and did not mislead observers.

#### 5.2.4 Results

**FIGURE 12:**
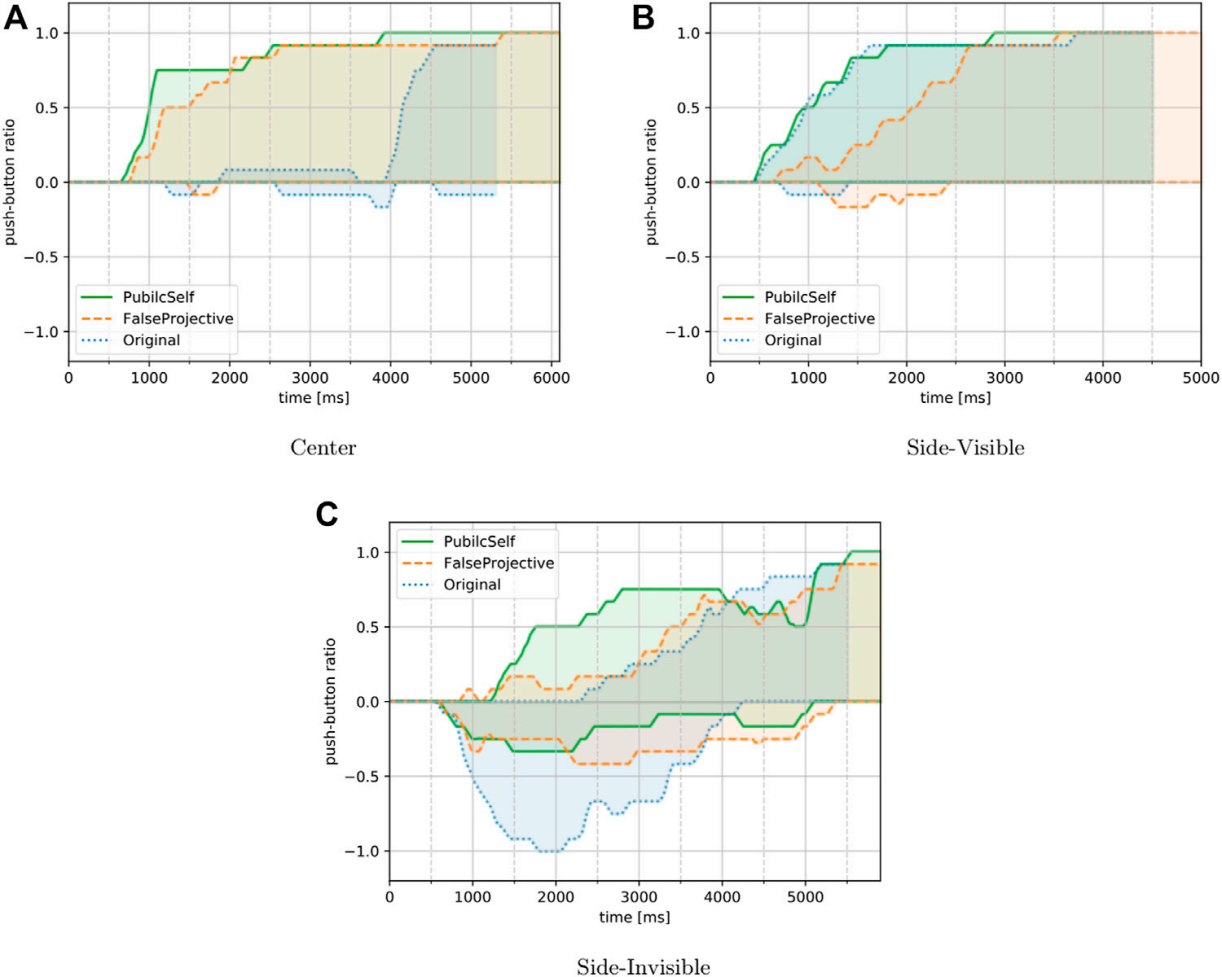
Participants’ inference. The upper lines show the percentages of participants whose answers were correct and indicate the rapidity of their inference. The lower shows incorrect cases, indicating the accuracy measure. PublicSelf enabled participants to both correctly and rapidly infer the actor’s intentions in the three episodes.**(A)**Center. **(B)**Side-Visible. **(C)**Side-Invisible.

Figure 12 shows the results for the participants’ inferences in the Center, Side-Visible, and Side-Invisible scenarios. Here, we adopted the number of correct answers as the measure of rapidity, and the number of wrong answers as the measure of accuracy. Tables 3, 4 summarize the results for our hypotheses.

**TABLE 3:** Results for the *rapidity* measure.

	Original	FalseProjective	PublicSelf
Center	✗	✓	✓
Side-Visible	✓	✗	✓
Side-Invisible	✗	✗	✓

**TABLE 4:** Results for the *accuracy* measure.

	Original	FalseProjective	PublicSelf
Center	✓	✓	✓
Side-Visible	✓	✓	✓
Side-Invisible	✗	△	△

Let us first focus on the rapidity measures. The results for the Center scenario were consistent with our expectations. The FalseProjective and PublicSelf legible motion trajectories could lead the participants to rapidly comprehend the actor’s true intention, while for the original motion, it was not until the actor turned toward its target that the participants comprehended the actor’s intention. The results in Side-Visible also supported our hypothesis. The FalseProjective legible motion failed to let participants infer the actor’s intention rapidly, while participants identified correct intentions more rapidly when provided original and PublicSelf motions. In Side-Invisible, PublicSelf allowed more rapid inference than original, but unexpectedly, FalseProjective could not improve rapidity. A possible explanation of this result is that participants became careful and held their judgments while watching FalseProjective motions. Two participants reported that they felt FalseProjective motions were somehow roundabout, while no participant mentioned PublicSelf detours. Such perception can delay a participant’s presentation of their judgment, resulting in less rapidity. In summary, 1) PublicSelf enabled human participants to rapidly infer the actor’s intention by considering information asymmetry between the actor and observer. 2) FalseProjective motion’s roundabout behavior seemed to make participants delay presentation of their judgments.

From the perspective of accuracy, few answers were wrong in the Center and Side-Visible scenarios for each motion type, and we find very few wrong answers for FalseProjective. This result is against our prior hypothesis but supports suggestion 2) in the last paragraph. That is, participants avoided wrong answers by delaying presentation of their judgment. In Side-Invisible, the original motion led to incorrect inferences, and all participants presented wrong judgments in 2,000 ms. On the other hand, the FalseProjective and PublicSelf motion trajectories did reduce the number of incorrect answers compared to the original motion, although some participants still had wrong answers. In summary, 3) FalseProjective and PublicSelf could reduce the number of wrong inferences compared to the original motion. 4) In terms of the accuracy of human observers’ inferences, we did not find differences between FalseProjective and PublicSelf.

**FIGURE 13:**
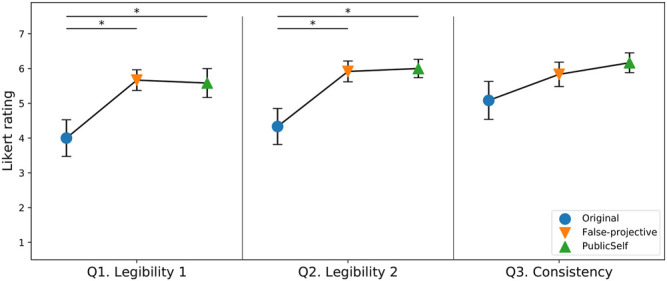
Subjective measures. The error bars indicate standard errors. Both FalseProjective and PublicSelf earned higher scores in the legibility measures, but no statistical difference was found between FalseProjective and PublicSelf.

Figure 13 shows the results for the subjective measures. The one-way repeated measures ANOVA showed that there were significant differences between the motion types for Q1 (*F*(2, 22) = 4.52, *p* < 0.05, *η* ^2^ = 0.21) and Q2 (*F*(2, 22) = 4.83, *p* < 0.05, *η* ^2^ = 0.26). For Q1 and Q2, post hoc Tukey tests revealed that the FalseProjective and PublicSelf legible motion trajectories were rated significantly higher than the original motion trajectories (*p* < 0.05). Two participants reported that the original motion in the Center scenario gave a strongly negative impression due to its illegibility. Contrary to our expectations, we did not find a significant difference between the FalseProjective and PublicSelf trajectories. Although two participants provided negative comments on FalseProjective’s roundabout behaviors in the Side-Invisible and Blind scenarios, it seems that their effects were limited compared to FalseProjective’s successful behavior in Center and Side-Visible. Similarly, the results did not show that the participants thought FalseProjective was inconsistent compared to the other motions. As a result, the subjective measures demonstrated participants’ positive perceptions toward both FalseProjective and PublicSelf motions.

#### 5.2.5 Summary of User Study

We found that legible motion generated with awareness of information asymmetry between an actor and an observer could successfully allow human observers to *rapidly* infer the actor’s intention compared to original motion, which does not consider human inference of the actor agent’s mind, and FalseProjective legible motion, which ignores information asymmetry. We did not find that FalseProjective motion misled human observers to wrong inferences, but the results suggest that FalseProjective’s roundabout behavior made the participants more cautious, which delayed their judgments.

## 6 Future Work

The legible motion generated with PublicSelf has been shown to be effective in the simple situations presented here, which indicates that given awareness of the information asymmetry between an artificial agent and a human observer, PublicSelf can successfully convey an actor’s certain intention. However, further evaluation is required to discuss the robustness of PublicSelf legible motion. Although the simulation study over 2,500 conditions is complementary to the user study with a small number of conditions, the data-driven artificial observers have an inference structure different from people, which can yield different results. For example, in our previous study (Fukuchi et al., 2018), people occasionally doubted the assumption that the actor intends to get either an apple or a pear and lost confidence on their answers, which never happens in simulation. Moreover, people can be affected by former trials while the artificial observers does not change after training phase. In this paper, we controlled such effect by randomizing the order of episodes and providing a small number of episodes, but to build an agent that can develop a long-term relationship with users, we need continuous experiments with more episodes. The small number and limited demographic variety of participants are also limitaions of the user study.

In addition, extensions of our method will be required for practical applications. One possible challenge is the calculations in PublicSelf. In this paper, we assumed the observer to be doing nothing other than observing the actor’s behavior. However, in an actual human-agent cooperation scenario, the humans involved also move, perform actions, and affect the environment, thereby making predictions of human observations and environmental transitions much more difficult. Another problem is the assumption of initial knowledge. The actor agent was assumed to always know the locations of the apple and pear, but in actuality, the actor, as well as the observer, will typically be subject to uncertainty. Although PublicSelf theoretically should also work in such situations, the strategy for exhibiting publicly self-aware behavior becomes more complex. For example, the actor must judge whether generating publicly self-aware behavior is possible in a given situation. Placing obstacles in an actor’s path can highlight interesing aspects of the strategy. It dramatically increases the complexity of an observer’s expectations about the environment or agents such as the area that an agent can perceive or possible paths that an actor can choose. An actor may need feedbacks to know such expectations or communication to align them. Tuning the thresholds for balancing the improvement of legibility and the pursuit of an actor’s true goal (Algorithm 1) was done by hand in this paper. However, it will be a problem because the best threshold can differ depending on the situation or the importance of conveying an intention against pursuing it. Reinforcement learning can be a promising approach to enable an actor to balance them automatically.

## 7 Conclusion

This paper focused on conveying an artificial agent’s certain intentions by motions. The main claim of this paper was that it is important to handle information asymmetry between an actor and its observer. To formalize this idea, we developed a method for generating motions that convey an agent’s intention with the awareness of information asymmetry using our previously proposed PublicSelf model. We conducted a simulation study and a user study to validate our claim. In both experiments, we focused on legible motion, which conveys an actor’s true intention to its observer. We compared PublicSelf’s legible motion with FalseProjective motion, which was generated without considering information asymmetry in an approach similar to those taken in previous studies. As a result, PublicSelf legible motion could successfully allow observers to quickly infer an actor’s intentions while FalseProjective sometimes compromised an observer’s predictions of an actor’s intentions in situations with information asymmetry. This result suggests that by considering information asymmetry, an agent can more effectively convey intentions with motions.

## Data Availability

The raw data supporting the conclusions of this article will be made available by the authors, without undue reservation.
